# Data Mining for Identification of Targets and Repurposed Drugs to Eliminate Persistent Chronic Myeloid Leukaemia Stem Cells: Targeting RAS/RAF Signalling

**DOI:** 10.32604/or.2026.074734

**Published:** 2026-04-22

**Authors:** I Made Bayu Anggriawan, Heather G. Jørgensen

**Affiliations:** 1 School of Medicine, Dentistry and Nursing, College of MVLS, University of Glasgow, Glasgow, UK; 2 Ngoerah General Hospital, Ministry of Health, Denpasar, Indonesia; 3 Lembaga Pengelola Dana Pendidikan (LPDP), Jakarta, Indonesia; 4 School of Cancer Sciences, College of MVLS, University of Glasgow, Glasgow, UK

**Keywords:** Chronic myeloid leukaemia (CML), leukaemic stem cells (LSCs), tyrosine kinase inhibitors (TKIs), RAS/RAF signalling pathway, PPP2CA, and drug repurposing/combination therapy

## Abstract

**Background:**

Persistent leukaemic stem cells (LSCs) in chronic myeloid leukaemia (CML) are insensitive to targeted tyrosine kinase inhibitors (TKIs). Identifying alternative molecular vulnerabilities may offer new therapeutic opportunities. This study aimed to identify active RAS/RAF signalling pathway components in persistent CML-LSCs using publicly available datasets to propose a novel drug combination that could synergise with TKI therapy.

**Methods:**

EMBL-EBI Single Cell Expression Atlas and Stemformatics were used to analyse gene expression within the chosen signalling pathway using DESeq2 analysis in R Studio. Genes that showed statistically significant differences across three comparisons (CML vs. normal; post vs. pre TKI; post TKI vs. normal) were evaluated for gene dependency (Chronos scores), expression profiles, and inhibitor sensitivity using the DepMap platform, with a focus on CML cell lines. Candidate inhibitors were identified using DrugBank.

**Results:**

*PPP2CA* demonstrated broad essentiality with negative Chronos scores consistent with strong gene dependency. Its expression was consistently high, reinforcing its biological relevance in CML. LB-100 was found as a PP2A inhibitor under trial. Sensitivity analysis revealed LB-100 affected 548 cancer cell lines broadly.

**Conclusion:**

*PPP2CA* represents a promising therapeutic vulnerability in CML, supported by both strong dependency and consistent expression in myeloid models, while BRAF showed limited relevance outside mutation-driven cancers. Variation in experimental platforms, sample representation, and data integration across public datasets is a recognised study limitation. Nonetheless, LB-100 may provide a novel therapeutic avenue in CML, provided further preclinical functional and clinical validation is performed in patient-derived samples to confirm translational applicability of the findings.

## 1 Introduction

Chronic myeloid leukaemia (CML) arises as a clonal myeloproliferative disorder caused by the Philadelphia chromosome, t(9;22), which forms the *BCR::ABL1* fusion gene. This gene produces a persistently active tyrosine kinase that underlies disease development by transforming normal haematopoietic stem cells (HSCs) into leukaemia stem cells (LSCs) [1]. The continuous activation of this kinase plays a critical role in disease progression, traditionally described as progressing from the chronic phase (CP) to the accelerated phase (AP), and ultimately to blast crisis (BC), where leukaemic proliferation becomes uncontrolled [1–3]. However, the World Health Organization (WHO) has recently revised the classification of CML to a biphasic disease, now recognising only the CP and BC phases [4]. The AP phase is no longer considered a distinct clinical stage due to evolving insights into disease progression and biological rationale.

Early treatment of CML was with chemotherapy such as hydroxyurea or interferon-alpha (IFNα), or allogeneic stem cell transplantation (allo-SCT). Hydroxyurea is an antimetabolite that targets ribonucleotide reductase (RNR), an enzyme that plays a crucial role in catalysing the reduction of ribonucleoside diphosphates into deoxyribonucleotide (dNTP) precursors, which are essential for DNA replication and repair. Inhibition of RNR significantly reduces cell proliferation. However, blocking these processes can induce a cellular senescence phenotype, which has been associated with therapy resistance, inflammation, immunosuppression, and cancer relapse. Currently, its use in CML is primarily limited to cytoreductive therapy—particularly in patients with hyperleukocytosis—as a pre-treatment before initiating tyrosine kinase inhibitors (TKIs) during CP-CML [5].

Furthermore, IFNα, an α-helical glycoprotein, initiates signalling by activating Janus kinases (JAKs), leading to the phosphorylation of signal transducer and activator of transcription (STAT) proteins [6,7]. These proteins promote the transcription of genes involved in antiproliferative and proapoptotic responses. IFNα also downregulates the expression of cyclin A, cyclin D3, cyclin E, and cdc25A, leading to decreased activity of the retinoblastoma protein (pRb) and inhibition of E2F release, which is essential for DNA replication [7,8]. Although IFNα has shown the ability to induce disease regression and improve survival, its use in clinical practice is restricted by its limited effectiveness and considerable toxicity. On the other hand, allogeneic stem cell transplantation (allo-SCT) remains a curative option, research has shown allo-SCT offers 60% increased 5 year survival rate in younger individuals with histocompatibility leukocyte antigen-matched siblings [9]. However, this approach carries high morbidity and mortality risks and can only be considered for individuals who have good overall condition, adequate organ function, and importantly access to a compatible stem cell donor [1].

The European Leukemia Net (ELN) guidelines recommend TKIs such as imatinib, as the first-line treatment for CML [10]. TKIs work by disrupting signalling pathways initiated by tyrosine kinases, including the epidermal growth factor receptor (EGFR). This disruption prevents downstream adaptor proteins like GAB1 (Grb2-associated binder) from recruiting PI3K, thereby halting phosphorylation processes. As a result, the MAPK pathway—comprising RAS, RAF, MEK, and ERK—can also be inhibited [11], which is crucial since this cascade plays a key role in promoting cellular proliferation and tumour development. Imatinib exerts its effects by competitively binding to the ATP-binding site of the BCR::ABL1 fusion protein, blocking tyrosine phosphorylation and effectively inhibiting the oncogenic signalling cascade [12]. Evidence from the International Randomized Study of Interferon and STI571 (IRIS) trial showed that daily administration of 400 mg imatinib significantly outperformed the combination of IFN-α and cytarabine across all key clinical outcomes [13]. Due to its pharmacological efficacy and favourable clinical results, imatinib has remained the first-line treatment for CML treatment for over twenty years.

Despite the success of TKIs, monotherapy is not curative for most patients due to the persistence of LSCs, which are often quiescent and not fully reliant on *BCR::ABL1* signalling. *Ex vivo* studies have suggested that the antiproliferative effect of TKIs may contribute to the induction of a quiescent state in LSCs, enabling them to evade TKI-mediated eradication [14,15]. In a single-cell analysis, Giustacchini et al. (2017) observed persistent LSC populations in CP-CML patients undergoing TKI therapy, characterised by a transcriptional profile associated with cellular quiescence [16]. Furthermore, in some cases, CML LSCs exhibit intrinsic resistance to TKIs, independent of *BCR::ABL1* expression. These LSCs may have low or undetectable levels of *BCR::ABL1* yet retain the ability to survive, further contributing to disease persistence [17]. LSC persistence enables evasion of eradication, contributing to minimal residual disease that in turn increases the risk of relapse, and potentially driving progression to BC-CML.

Most patients with CML are diagnosed during the relatively stable CP, which is often asymptomatic or presents with mild symptoms and is characterized by fewer than 10% blast cells in the bone marrow or blood. However, without effective treatment, CML will progress to the more aggressive BC, either myeloid or lymphoid in lineage [18]. In this terminal stage, the disease mirrors acute leukaemia, marked by a rapid increase in immature blast cells (≥20%), genomic instability, suppression of normal blood cell production, and poor prognosis [10]. BC-CML shares several clinical and biological features with acute myeloid leukaemia (AML), including high proliferative activity, resistance to standard therapy, and short median survival, making early diagnosis and intervention during CP critical to preventing disease progression.

**Figure 1:**
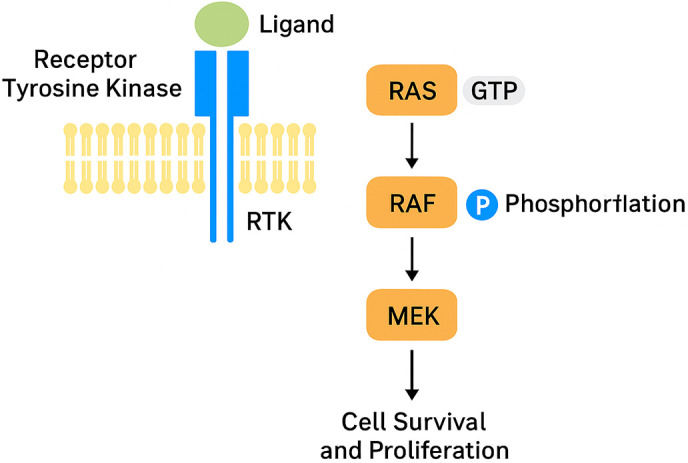
RAS/RAF signaling on proliferation and anti-apoptosis. Abb: Receptor Tyrosine Kinase (RTK), Rat Sarcoma (RAS), Rapidly Accelerated Fibrosarcoma (RAF), Mitogen-Activated Protein Kinase Kinase (MEK).

The RAS/RAF–MAPK/ERK signalling pathway typically transmits growth-promoting and survival signals from membrane receptors to intracellular targets, and its dysregulation is strongly implicated in myeloid malignant transformation [19,20]. This pathway is activated through the interaction between ligands and the extracellular domain of receptor tyrosine kinases (RTKs) [21,22]. Upon GTP binding, RAS activates RAF by translocating it to the plasma membrane. RAF then stimulates a kinase cascade involving MEK and ERK, producing downstream signals that enhance proliferation and prevent apoptosis (Fig. 1) [23].

Extensive evidence from both cellular and animal studies highlights the RAS/ERK signalling cascade as a crucial mediator of *BCR::ABL1*–driven leukaemogenesis. This has led to significant interest in understanding how *BCR::ABL1* activates the pathway. One proposed mechanism involves the recruitment of the guanine nucleotide exchange factor (GEF) Son of Sevenless (SOS) through adaptor proteins such as Grb2 and Gab2, facilitating Ras activation [24]. Interestingly, a study by Hentschel et al. (2011) uncovered a mechanism of imatinib resistance that does not depend on *BCR::ABL1* activity. In resistant CML cells, persistent activation of the ERK-MAPK pathway was observed despite no detectable changes in *BCR::ABL1* or RAS function. The sustained signalling was attributed to constitutive c-RAF activity, which continued to drive proliferation independently of *BCR::ABL1* inhibition [25]. These findings imply that RAF signalling can support leukaemic cell survival under TKI pressure, suggesting that direct targeting of RAF may offer an alternative strategy to combat TKI resistance in CML.

Sorafenib is a well-known RAF inhibitor that blocks MAPK pathway activation by targeting its upstream regulator, RAF kinase [26]. It is a multikinase inhibitor with activity against several key tyrosine kinases, including FMS-like tyrosine kinase 3 (FLT3), vascular endothelial growth factor receptor 2 (VEGFR-2), c-KIT, and RET. Sorafenib has been used in the treatment of AML [27], largely due to its inhibitory effects on FLT3, a kinase critical for the proliferation of haematopoietic stem and progenitor cells. FLT3 alterations—particularly internal tandem duplications (ITDs) and D835 point mutations—are found in approximately 20%–30% of adult AML cases and are often correlated with adverse outcomes [26]. Given sorafenib’s ability to simultaneously inhibit both RAF and FLT3 signalling pathways, it presents a promising candidate for repurposing in CML, particularly in addressing LSC persistence/TKI insensitivity driven by alternative survival pathways.

This study aims to identify active RAS/RAF signalling components in TKI-persistent CML-LSCs using publicly available datasets and to propose a novel drug combination that could synergise with TKI therapy. To investigate relevant gene pathways, three different comparisons were planned using single-cell transcriptomic data: (1) normal HSCs vs. CML-LSCs, (2) CML stem cells before vs. after TKI treatment, and (3) CML stem cells after TKI treatment vs. normal HSCs. Based on these novel insights from the literature, a new candidate gene target may be proposed that, in combination with imatinib, could enhance therapeutic efficacy in CML.

## 2 Materials and Methods

### 2.1 Single-Cell RNA-Seq Analysis

Publicly available single-cell RNA sequencing datasets (freely available at ebi.ac.uk/gxa/sc/home) were analysed to identify signaling pathways that remain active in CML patients’ LSCs, particularly during and after TKI therapy. Processed single-cell RNA-seq data from Giustacchini et al. (2017) (E-GEOD-76312) were downloaded from the EMBL-EBI Single Cell Expression Atlas [16,28]. The Giustacchini et al. dataset includes single-cell transcriptomes from normal donors and CML patients sampled across multiple disease and treatment time points. LSCs were defined based on the original immunophenotypic annotations of primitive hematopoietic stem cell populations. We used the authors’ preprocessed matrices, which include quality-controlled and normalized gene-by-cell counts, annotated cell populations, and UMAP embeddings. As described in the original publication, the preprocessing pipeline consisted of filtering low-quality cells, normalization, identification of highly variable genes, PCA dimensionality reduction, and clustering, followed by UMAP visualization. Therefore, there were no re-running QC, clustering, or dimensionality-reduction steps conducted as the original preprocessing was deemed sufficient for the specific downstream analyses performed here.

Validation analyses were performed at the candidate gene level using independent CML datasets from Gerber et al. (2013; GSE43754) and Cramer-Morales et al. (2013; GSE47927), available through the Stemformatics platform (freely available at https://www.stemformatics.org/) [29], were additionally analyzed. For each candidate gene identified in the primary EMBL-EBI (Giustacchini et al.) analysis, gene expression profiles were queried using the Stemformatics’ genes feature. Validation was based on assessing the concordance in the direction of expression changes between CML and normal samples, rather than re-performing genome-wide differential expression analyses.

A targeted, pathway-driven gene set was defined prior to differential expression analysis to focus on mechanisms implicated in CML persistence. Genes associated with RAS/RAF signaling were identified using a two-step, systematic curation approach. First, all genes annotated as members of the RAS/RAF and downstream MAPK signaling pathways in the KEGG database, freely available here: www.genome.ad.jp/kegg/ (hsa04010: MAPK signaling pathway; hsa04014: Ras signaling pathway), were reviewed [30] and pathway-relevant genes were listed individually based on their established roles in signal transduction. This approach was used to ensure biological relevance while maintaining transparency in gene selection.

Second, this list was cross-referenced with genes reported in prior experimental and mechanistic studies investigating BCR-ABL–independent survival and imatinib resistance in CML stem cells, including Steelman et al., Bahar et al. and Ma et al. [31–33]. Genes were retained if they were repeatedly reported to modulate RAS/RAF pathway activity, MAPK signaling output, or leukemic stem cell survival. This approach was intended to prioritize mechanistically relevant genes rather than to provide an exhaustive representation of all pathway members.

### 2.2 Statistical Analysis of RNA-Seq Data

**Figure 2:**
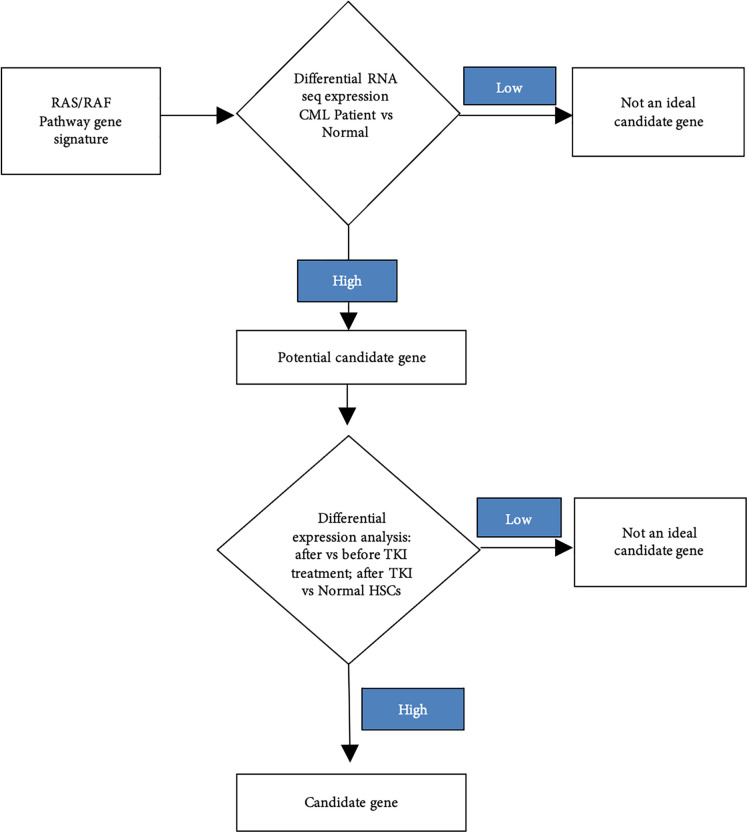
Flow chart of finding gene candidate. Abb: chronic myeloid leukaemia (CML), tyrosine kinase inhibitor (TKI), haematopoietic stem cell (HSC).

DESeq2 was selected for differential expression analysis because it is specifically designed for count-based RNA-seq data and uses a negative binomial model with empirical Bayes shrinkage, providing robust dispersion and fold-change estimates, particularly in studies with limited sample sizes [34,35]. All analyses were performed in R using RStudio (v 4.5.2) [36]. Processed gene expression matrices and associated metadata were imported from the Giustacchini et al. dataset and reorganized for the purpose of normal donor- vs. CML patient, before–after TKIs treatment, and after TKIs treatment- normal donor comparison. The analytical workflow for this data mining step is illustrated in Fig. 2.

The original metadata included multiple sampling timepoints (3, 6, 12, 18, and 60 months of TKI treatment), “diagnosis”, “before blast crisis”, “blast crisis”, and a “not applicable” category. For the purpose of differential expression and comparative analysis, we redefined these into biologically relevant groups: Normal controls: “not applicable”, and CML Patient (before TKIs treatment): “at diagnosis”. For CML patients after TKI treatment, samples collected at 3, 6, and 12 months were pooled to represent an early treatment phase characterized by sustained BCR-ABL1 inhibition and initial molecular adaptation, while samples at 18 and 60 months were excluded due to limited sample size. We acknowledge that this pooled group may encompass transcriptional heterogeneity reflecting differences in the depth and kinetics of treatment response across time points. However, pooling was necessary to ensure adequate sample size and statistical power and to capture shared treatment-associated transcriptional features relevant to early TKI persistence. Cell identities (stem/progenitor populations) were kept as defined in the original annotation [37].

Counts were subset to samples present in the filtered metadata, and metadata were reordered to ensure one-to-one correspondence with expression columns. Low-expression genes were removed using a row-sum threshold of >10 to reduce noise and improve statistical power [38]. A DESeq2 object was then constructed using the specified design formula, and differential expression analysis was performed using DESeq2’s Wald test with default settings. No external normalization, scaling, or QC corrections were applied, as DESeq2 performs internal normalization via size-factor estimation. Genes were classified as upregulated or downregulated based on adjusted *p*-value < 0.05 and |log2 fold-change(FC)| > 1 [34]. All results, including MA plots and annotated volcano plots, were exported for reproducibility. The full analysis script and session information are provided in Supplementary Materials S1.

### 2.3 Literature-Driven Pathway

A literature review was conducted to support and validate the transcriptomic findings. Recent review articles and primary research publications in the field of CML stem cell biology and TKI resistance were examined. Particular attention was given to studies discussing the molecular mechanisms underlying LSC survival, self-renewal, and resistance to targeted therapies.

For each gene or pathway identified in the single-cell analysis, supporting evidence from the literature was compiled to confirm its functional role in LSC persistence. The potential for therapeutic intervention was assessed by evaluating whether the target had previously been explored in drug development. Based on the integration of transcriptomic data and literature evidence, a shortlist of biologically relevant and potentially druggable targets was generated.

### 2.4 Drug Target Identification

Drug target identification was carried out for all the shortlisted genes and pathways meeting the predefined criteria. Existing pharmacological agents with inhibitory activity against these targets were mined using the DrugBank database version 5.1.13, freely available at go.drugbank.com [39]. For each target, drugs with documented inhibitory activity were retrieved and prioritized based on the following criteria: (i) mechanism of action consistent with pathway inhibition, (ii) evidence of target specificity or primary target engagement, (iii) existing clinical indication, with preference for FDA-approved or clinically investigated agents, and (iv) reported toxicity and safety profiles derived from clinical use or trial data. This information was qualitatively integrated to assess biological relevance and feasibility for drug repurposing rather than to generate a quantitative ranking.

**Figure 3:**
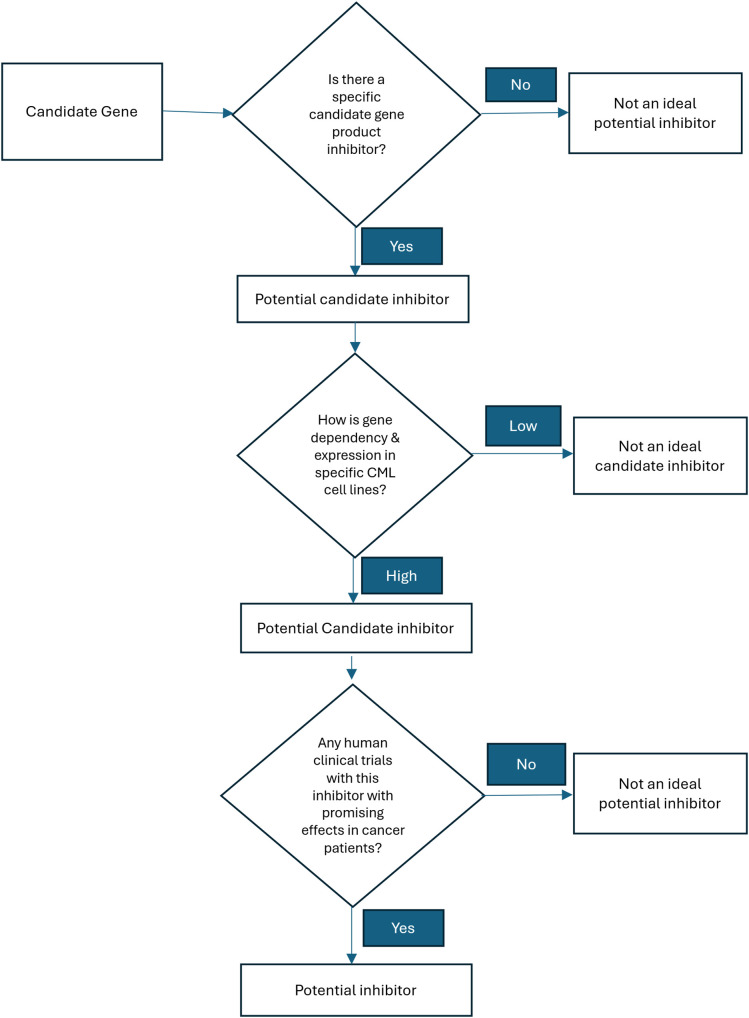
Flow chart of finding inhibitor for novel drug combination.

To evaluate the functional importance of each candidate gene, dependency data were extracted from the Cancer Dependency Map (DepMap Public 25Q2) [40]. Dependency scores and gene essentiality data derived from CRISPR-Cas9 and RNA interference (RNAi) screens in CML-related cell lines were reviewed. This analysis helped prioritise targets whose inhibition was predicted to compromise leukaemic cell survival. Targets that were both essential for CML cell survival and actionable by existing drugs were considered strong candidates for further preclinical or translational investigation. This strategy was employed to explore the potential of rational drug repurposing, particularly in combination with TKIs, with the aim of enhancing therapeutic efficacy and overcoming resistance mechanisms in CML. The workflow for the drug identification step is illustrated in Fig. 3.

The candidate gene inhibitors were further evaluated using the IDACombo platform [41], which applies an independent drug action (IDA)–based approach to predict the efficacy of drug combinations [42]. Single-agent drug response data were loaded from the Cancer Therapeutics Response Portal version 2 (CTRPv2), which provides broad coverage across diverse cancer cell lines. To ensure disease relevance, analyses were restricted to hematopoietic and lymphoid malignancy–derived cell lines using the platform’s built-in filtering option. Candidate inhibitors corresponding to the shortlisted target genes were selected as single agents, and predicted combination effects were calculated using the platform’s default Independent Drug Action (IDA) model by computing IDACombo scores and associated hazard ratios. No additional parameter tuning was applied. Predicted drug combinations were exported and further processed in R (v4.3.x), where combinations were ranked, and the top 30 candidate pairs were selected and reformatted to prioritize those with the highest predicted combinatorial efficacy and examined for potential synergistic effects.

## 3 Results

### 3.1 Single Cell RNA-Seq Analysis

The publicly available dataset GSE76312 was analysed, which comprises over 2000 single-cell RNA sequencing profiles from 27 CML patients at diagnosis and after 3 to 60 months of TKI therapy, along with samples from 6 healthy patients as controls. This analysis aimed to explore potential vulnerabilities in the persistent LSC population. The genes analysis was focus on the RAS/RAF signaling pathway; there were 48 genes included in the study based on Bahar et al. study [32] and the KEGG pathway database [30].

Differential expression was assessed using DESeq2 by comparing the cell populations described in Table 1 and detail of the 48 genes is presented in the Supplementary Material Tables S1–S3. A total of seventeen genes showed significant expression differences between normal HSCs and CML patients, thirteen genes were significantly altered in CML patients before vs. after TKI treatment, and twelve genes were differentially expressed between normal HSCs and CML patients following TKI therapy (Table 1).

**Table 1:** Differential gene expression profiles from single-cell sequencing of CML patient cells.

Comparison	Significant Differentiation (*p*-adj Value < 0.05)			Not Significant	Total Genes Analysis
	Upregulated	Downregulated	Total		
CML patients vs. normal donor	17	0	17	31	48
After vs. before TKIs treatment	3	10	13	35	48
After TKIs treatment vs. normal donor	9	3	12	36	48

Note: Abb: Chronic myeloid leukaemia (CML). https://www.ebi.ac.uk/gxa/sc/experiments/E-GEOD-76312/results/cell-plots.

Candidate genes were selected based on upregulation in each comparison, prioritising those with the lowest adjusted *p*-values and highest fold changes. In the analysis, PPP2CA was significantly upregulated across all comparisons: CML diagnosis vs. normal patients, CML patients before vs. after TKI treatment, and normal patients vs. CML patients after TKI treatment (3–12 months therapy-free remission), with log2 fold changes of 1.14 (adj. *p* = 2.2 × 10^−4^), 1.11 (adj. *p* = 3.1 × 10^−3^), and 3.57 (adj. *p* = 7.4 × 10^−11^), respectively.

Similarly, BRAF showed significant upregulation when compared with normal patients (HSCs), with log2 fold changes of 1.79 (adj. *p* = 6.1 × 10^−12^) in CML diagnosis vs. normal, and 2.69 (adj. *p* = 1.1 × 10^−8^) in normal vs. CML patients after TKI treatment. No significant change was observed in BRAF expression between CML patients before and after TKI treatment (log2FC = 0.22, adj. *p* = 0.583).

**Figure 4:**
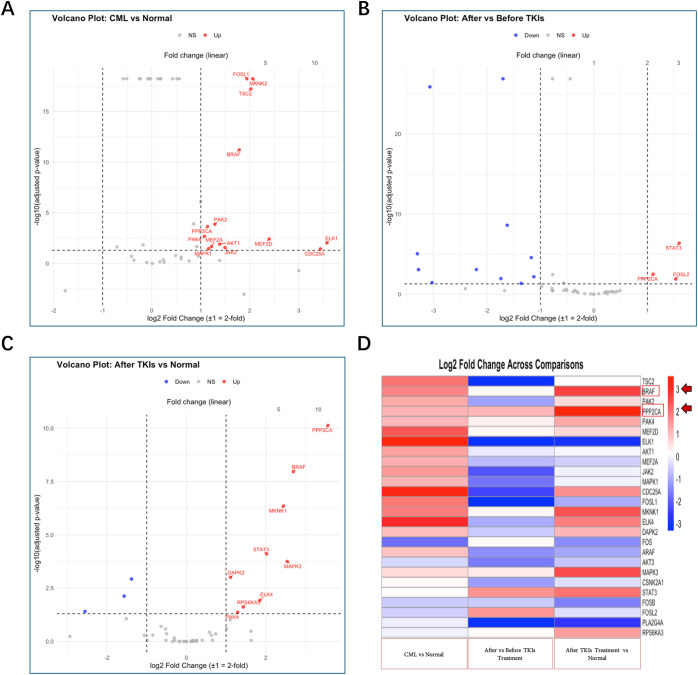
Gene regulation across three differential expression analyses. (**A**): chronic myeloid leukaemia (CML) vs. normal, (**B**): after vs. before tyrosine kinase inhibitor (TKI) and (**C**): after TKI vs. normal. Regulation was determined using a two-fold change threshold: genes with log2 fold change > 1 were classified as upregulated (red), while those with log2 fold change < −1 were classified as downregulated (blue). Genes with log2 fold change values between −1 and 1 were considered not significant, and genes with adjusted *p*-values (padj) > 0.05 were also classified as not significant (grey). PPP2CA was consistently upregulated across all three analyses, while BRAF showed consistent upregulation in CML patient comparisons. (**D**): differential expression was visualised in the heatmap, where red indicates upregulated genes and blue indicates downregulated genes. PPP2CA and BRAF were prominently upregulated (strong red signal) in the comparisons involving CML patients and healthy normal controls; rows are highlighted by red boxes and arrows.

PAK4 followed a similar pattern to BRAF but with lower log2 fold changes. Moreover, STAT3 was upregulated in CML patients before vs. after TKI treatment and in normal vs. CML patients after TKI treatment (3–12 months therapy-free remission); however, its expression levels remained lower compared with PPP2CA and BRAF. Details are shown in Fig. 4, with full data provided in Supplementary Tables S1–S3.

### 3.2 PPP2CA and BRAF as Candidate Genes

**Figure 5:**
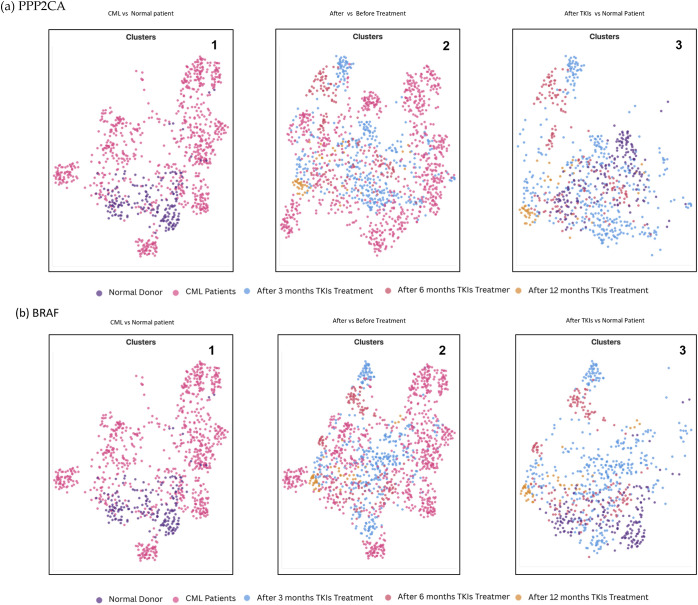
PPP2CA and BRAF time-point-based clustering across different comparation. (**a**) PPP2CA: (**a1**) CML at diagnosis vs. normal donor, showing clear separation between leukemic and healthy populations; (**a2**) before vs. after TKI treatment (3–12 months), where partial reorganisation toward normal-like clusters is observed but residual abnormal populations persist; (**a3**) after TKI treatment vs. normal HSCs, showing incomplete convergence to normal profiles. (**b**) BRAF: (**b1**) CML at diagnosis vs. normal donor, with distinct clustering of leukaemic cells; (**b2**) before vs. after TKI treatment, where clustering shows limited reorganisation and persistence of abnormal subpopulations; (**b3**) after TKI treatment vs. normal HSCs, demonstrating sustained divergence from normal clusters. UMAP visualizations were obtained directly from the EMBL-EBI Single Cell Expression Atlas for the dataset published by Giustacchini et al., and were used for descriptive and illustrative purposes only.

Clustering based on sampling time points highlighted consistent differences across disease stages and treatment for both PPP2CA and BRAF analyses. At diagnosis, CML patient samples formed clusters clearly separated from normal HSCs, reflecting transcriptional reprogramming characteristic of the leukaemic state. In the comparison between pre- and post-TKI treatment (3–12 months), clustering patterns showed partial reorganisation, with treated samples beginning to diverge from diagnosis-associated clusters and moving closer to normal profiles. However, in both PPP2CA- and BRAF-related analyses, distinct residual clusters persisted, suggesting incomplete transcriptional recovery (Fig. 5).

When post-TKI samples were compared directly with normal HSCs, convergence towards normal-like clustering was observed but not complete. For PPP2CA, treated samples showed partial restoration with some clusters resembling normal patterns, whereas BRAF-associated clustering remained more distinctly separated, indicating reduced sensitivity to treatment-induced transcriptional reprogramming. Taken together, these findings suggest that while TKI therapy can partially reshape the cellular landscape, persistent transcriptional abnormalities remain, with PPP2CA reflecting a more treatment-responsive trajectory and BRAF representing a more stable, resistant signature that may contribute to residual disease (Fig. 5).

**Figure 6:**
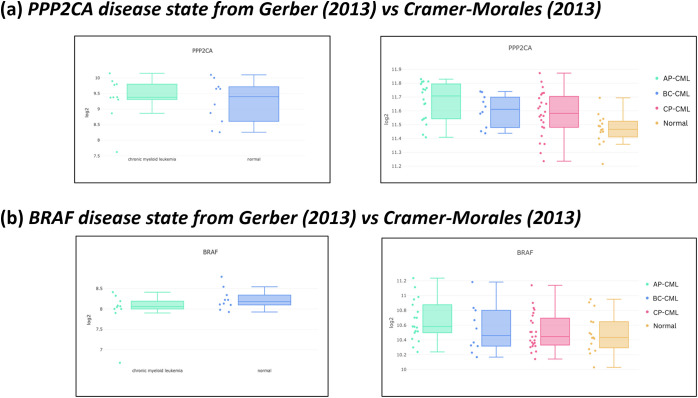
Expression of PPP2CA and BRAF across two independent datasets accessed via Stemformatics with Gerber et al. (2013) shown on left and Cramer-Morales et al. (2013) on right. The Gerber dataset is divided into two clusters (chronic myeloid leukaemia, CML (green) vs. normal (blue)), whereas the Cramer-Morales dataset is classified into four groups (accelerated phase (AP)-CML in green, blast crisis (BC)-CML in blue, chronic phase (CP)-CML in red, and normal in yellow). It is important to note that the current WHO classification recognises CML as a biphasic disease (CP-CML and BC-CML). (**a**): left panel shows PPP2CA expression in CML (green) vs. normal (blue) from Gerber et al. (2013); right panel shows differences in PPP2CA amongst CML disease phases and normal from Cramer-Morales et al. (2013). (**b**): left panel shows BRAF expression in CML (green) vs. normal (blue) from Gerber et al. (2013); right panel shows differences in BRAF amongst CML disease phases and normal from Cramer-Morales et al. (2013).

Validation of PPP2CA and BRAF expression was performed using two independent microarray datasets. The Gerber et al. (2013) dataset included 20 samples across 3 cell types (GSE43754) [43], allowing comparison of CML and normal states. Analysis showed that PPP2CA expression was elevated in CML compared with normal controls, confirming its role in the leukaemic state (Fig. 6a). BRAF expression, however, showed less pronounced differences, with only modest separation between CML and normal samples (Fig. 6b).

The Cramer-Morales et al. (2013) dataset included a larger cohort of 67 samples spanning 5 cell types (GSE47927) [44], covering multiple CML disease phases (chronic, accelerated, and blast phase) as well as normal controls. PPP2CA remained consistently expressed at higher levels across all CML phases compared with normal samples, indicating a stable dysregulation throughout disease progression (Fig. 6a). In contrast, BRAF expression displayed variability between phases, with sustained but less uniform expression differences compared with normal samples (Fig. 6b).

These findings strengthen the evidence for PPP2CA as a robustly upregulated gene in CML, validated across independent studies with different cohort sizes and cell types. Its consistent elevation across both Gerber (2013; 20 samples, 3 cell types) and Cramer-Morales (2013; 67 samples, 5 cell types) underscores its potential as a therapeutic vulnerability persisting throughout disease progression. BRAF, while detectable across both datasets, demonstrated less consistent patterns.

### 3.3 PPP2CA and BRAF Genes Dependency and Expression

Gene dependency and expression were reviewed using DepMap portal by Broad Institute. PPP2CA (Protein phosphatase 2 catalytic subunit alpha) shows strong overall dependency, with 1040/1183 CRISPR cell lines classified as strongly selective and a median Chronos score of around −1, reflecting broad essentiality across cancers. Importantly, this dependency extends to myeloid lineages, confirming its relevance within haematological contexts such as CML (Table 2). Gene expression analysis demonstrates broadly elevated levels (log2TPM + 1: 4–8) across tissues, with consistently high expression also observed in myeloid cell lines, highlighting its biological importance in both solid and blood cancers. Although no FDA-approved inhibitors exist, PPP2CA remains tractable as an enzymatic target, with the experimental inhibitor LB-100 under study.

**Table 2:** Functional and lineage characteristics of PPP2CA and BRAF in cancer models.

Category	PPP2CA	BRAF
Full Name	Protein phosphatase 2 catalytic subunit alpha	B-Raf proto-oncogene, serine/threonine kinase
Dependent Cell Lines	CRISPR: 1040/1183 (Strongly selective, Common essential); RNAi: 184/708	CRISPR: 93/1183 (Strongly selective); RNAi: 45/712
CRISPR Chronos Gene Effect	- Median ~ −1 (strong dependency overall); consistently negative across cancers. - Showing an essential in myeloid lineage with Chronos score around −1 (n = 43).	- Median ~ −0.5 (not strongly essential overall); highly negative in melanoma, thyroid, colon. - Near neutral (≈ −0.3 to −0.5)/weak dependency in myeloid lineage
Enriched Lineages (CRISPR)	Pancreatic adenocarcinoma, Breast, Pancreas, Lymphoid, Haematopoietic + Lymphoid, Solid, Uterus	Melanoma, Skin, Thyroid, Colon adenocarcinoma, Bowel, Lung
Expression	- Overall, log2TPM + 1: Higher (4–8); broadly expressed across cancers; high in breast, pancreas, lymphoid - Moderate–high expression; consistently expressed → essential in myeloid lineage (n = 73)	- Moderate (2–6); high in melanoma, thyroid, colon; lower in fibroblast/muscle - Moderate expression; not a main driver in myeloid cancers (n = 73)
Target Tractability	gene is enzymatic in nature and has a well-defined, druggable structure, with bioactive compounds already available but do not have a potential to be targeted by ligand-based drug discovery approaches	gene is enzymatic in nature and has a well-defined, druggable structure, with bioactive compounds already available and have a potential to be targeted by ligand-based drug discovery approaches
Drugs/Compounds	No EMA/FDA-approved inhibitors; experimental LB-100 (PP2A inhibitor, in trials)	Multiple EMA/FDA-approved inhibitors: Dabrafenib,Vemurafenib, Encorafenib

RAF (B-Raf proto-oncogene, serine/threonine kinase) shows narrower dependency, with 93/1183 CRISPR cell lines and 45 RNAi cell lines classified as strongly selective, and an overall median Chronos score of around −0.5, indicating more context-specific essentiality. Within myeloid lineages, BRAF retains selective dependency, suggesting a role in haematological malignancies in addition to its established role in melanoma, thyroid, and colorectal cancers. Expression is moderate overall (log2TPM + 1: 2–6), but higher in tumour types such as melanoma and thyroid, while myeloid cell lines also demonstrate detectable expression, supporting its potential contribution to signalling in CML contexts. Unlike PPP2CA, BRAF is already druggable, with several FDA-approved inhibitors available, including dabrafenib, vemurafenib, and encorafenib.

**Figure 7:**
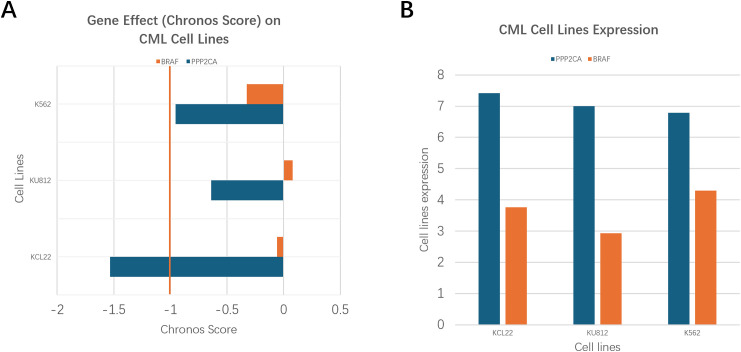
Comparison of PPP2CA and BRAF dependency and expression in CML cell lines. (**A**) Gene effect (Chronos scores) from DepMap CRISPR screens, where lower values indicate stronger essentiality (−1 = median score of pan-essential genes). PPP2CA shows strong dependency across KCL22, KU812, and K562, with KCL22 exceeding the pan-essential threshold. BRAF scores remain close to zero, indicating limited dependency. (**B**) Gene expression (log2TPM) demonstrates consistently higher expression of PPP2CA compared with BRAF across the three CML cell lines.

In the analysis of the specific myeloid-derived CML cell lines (KCL22, K562, and KU812), PPP2CA showed strong functional dependency (Fig. 7A) and consistent expression (Fig. 7B). Chronos scores indicated high essentiality with values of −1.53 in KCL22, −0.95 in K562, and −0.64 in KU812, all below zero, confirming that PPP2CA contributes to cell survival. Notably, KCL22 scored lower than −1, suggesting dependency stronger than the median for pan-essential genes. Correspondingly, PPP2CA expression levels were elevated across the three lines, with log2TPM values between 6.79 and 7.41, confirming active transcription that aligns with its dependency profile.

By contrast, BRAF displayed weaker dependency in the same cell lines, with Chronos scores of −0.40 (KCL22), −0.30 (K562), and −0.25 (KU812), values closer to zero and above the −1 pan-essential threshold, suggesting limited or context-dependent essentiality. Expression levels of BRAF were also lower than PPP2CA, with log2TPM values between 4.2 and 5.1, confirming moderate transcriptional activity in myeloid contexts.

### 3.4 Identification of Drug Target

#### 3.4.1 Candidate Genes Inhibitor

**Figure 8:**
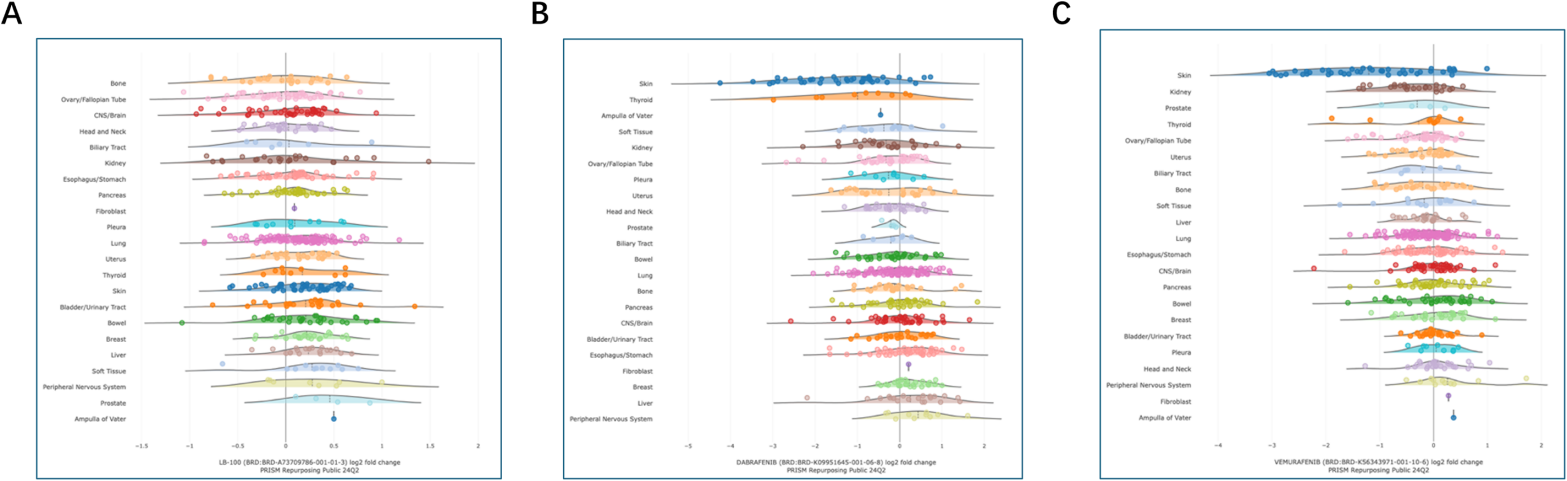
Lineage-specific drug sensitivity of LB-100 (**A**), dabrafenib (**B**), and vemurafenib (**C**) in the PRISM repurposing public 24Q2 dataset. Each panel summarizes log2 fold-change (LFC) in viability for individual cancer cell lines grouped by lineage at the assayed concentration(s). Dots are single cell lines; the gray curve depicts the smoothed distribution for that lineage. The vertical reference line at 0 marks no effect; negative LFC values indicate decreased viability (greater sensitivity), whereas positive values indicate relative resistance or growth. Short horizontal ticks mark the central tendency for each lineage (median), with the adjacent thin bars indicating spread. Notably, dabrafenib and vemurafenib show left-shifted distributions in skin lineages—consistent with sensitivity in BRAF-mutant melanoma—while LB-100 exhibits a broader, lineage-agnostic spread with fewer strongly left-shifted clusters.

LB-100 has been reported to influence PPP2CA expression in various malignancies and is currently the only PP2A inhibitor tested in a Phase I clinical trial [45]. In contrast, vemurafenib and dabrafenib are selective BRAF inhibitors that are widely used in the treatment of melanoma [46,47]. Both agents have also been evaluated in hairy cell leukemia, supporting their potential utility beyond solid tumours [48,49]. Therefore, these compounds were selected as candidate drugs for repurposing in combination with TKI therapy to target CML persistence. The comparison of inhibitors was analysed using DepMap database, highlighting clear differences between PPP2CA- and BRAF-targeting agents. LB-100, an investigational small molecule, directly inhibits PPP2CA, the catalytic subunit of PP2A, thereby modulating phosphatase activity. Sensitivity data show that LB-100 affects a broad range of cancer models, with responses observed in 548 cell lines, suggesting widespread essentiality (Table 3). In contrast, the BRAF inhibitors dabrafenib and vemurafenib are clinically approved small molecules that act as ATP-competitive inhibitors of mutant BRAF, particularly the V600E variant. Their activity is more lineage-restricted, with dabrafenib showing sensitivity in 221 cell lines enriched for melanoma and thyroid cancers, and vemurafenib in 254 cell lines, also primarily melanoma-enriched (Table 3). Notably, none of these candidate inhibitors has been tested in myeloid cell lines (Fig. 8), including CML models, leaving their relevance in this context undetermined.

**Table 3:** Overview of PPP2CA and BRAF inhibitors.

Description	PPP2CA Inhibitor	BRAF Inhibitor	
	LB-100	Dabrafenib	Vemurafenib
DrugBank ID	DB15412	DB08912	DB08881
Primary Target/Mechanism	Inhibits PPP2CA (PP2A catalytic subunit)—phosphatase inhibitor	ATP-competitive inhibitor of mutant BRAF (V600E/K/D) → blocks MAPK pathway	Competitive inhibitor of BRAF V600E → inhibits MAPK signalling
DepMap Sensitivity	548 cell lines (broad sensitivity, essential target)	221 cell lines (selective, melanoma/thyroid enriched)	254 cell lines (selective, melanoma enriched)
Enriched Lineages (PRISM/DepMap)	Broad, no strong lineage-specific enrichment	Melanoma (*p* = 7.55 × 10^−25^, n = 36) Skin (7.55 × 10^−25^) n = 36	Melanoma (*p* = 1.85 × 10^−26^, n = 32) Skin (1.85 × 10^−26^) n = 32
Pharmacodynamics	Not available	Reduces MAPK activity, often combined with trametinib to delay resistance	Reduces MAPK/ERK activity, almost complete MAPK inhibition in sensitive tumours
Pharmacokinetics	Not available	Oral, Tmax = 2 h; bioavailability 95%; half-life = 8 h; metabolised by CYP2C8 & CYP3A4; fecal (71%) + renal (23%) excretion	Oral, Tmax = 3 h; half-life = 57 h; metabolised mainly by CYP3A4; excreted mostly in feces (94%)
Protein Binding	Not available	99.7% bound	>99% bound
Adverse Effects/Toxicity	Not available	Cutaneous squamous cell carcinoma risk; fertility effects in animals; drug–drug interactions via CYP	Cutaneous squamous cell carcinoma, paradoxical tumour growth, photosensitivity
Food/Drug Interactions	Not reported	Grapefruit & St John’s Wort; high-fat meals; take on empty stomach	High-fat meals (↑ exposure ×5); grapefruit (↑ exposure); caffeine & St John’s Wort (hypericum perforatum)

According to DrugBank, pharmacodynamic and pharmacokinetic data for LB-100 remain limited, whereas detailed information is available for the approved BRAF inhibitors dabrafenib and vemurafenib (Table 3). Pharmacokinetic profiling highlights differences between the two agents: dabrafenib is rapidly absorbed (Tmax ≈ 2 h) with a short half-life (~8 h), while vemurafenib shows slower absorption (Tmax ≈ 3 h) and a prolonged half-life (~57 h). Both agents are extensively protein-bound (>99%) and are primarily metabolised by CYP3A4; however, dabrafenib additionally involves CYP2C8 and is excreted via faeces (71%) and urine (23%), whereas vemurafenib is eliminated almost exclusively through faeces (94%). Despite overlapping adverse effects such as cutaneous squamous cell carcinoma, photosensitivity, and paradoxical tumour growth, dabrafenib is more frequently associated with pyrexia and metabolic drug interactions, while vemurafenib carries an additional risk of QT prolongation. These findings emphasise the investigational but broad potential of LB-100, in contrast to the clinically established yet mutation-restricted role of BRAF inhibitors.

LB-100 is currently investigational and undergoing Phase I/II trials in glioblastoma, ovarian clear cell carcinoma, colorectal cancer, and myelodysplastic syndromes [45]. It is classified as an antineoplastic enzyme inhibitor and is notable for its novel mechanism, broad dependency, and potential synergy with chemotherapy and immunotherapy (Table 4). However, its limitations include systemic toxicity, given its action on a common essential gene, lack of selectivity, and its early-stage development status. In contrast, the BRAF inhibitors dabrafenib and vemurafenib are already FDA/EMA/MHRA approved. Dabrafenib, approved in 2013, is indicated for melanoma (V600E/K), NSCLC (V600E), thyroid cancer, paediatric glioma, and other solid tumours. It is classified under L01EC02 (BRAF inhibitors) and has shown strengths in precision oncology, being highly effective for BRAF-mutant tumours with strong data in combination with trametinib. Its limitations are centred on resistance due to MAPK reactivation and lack of efficacy in wild-type BRAF or colorectal cancer. Vemurafenib, approved earlier (2011–2012), is indicated for melanoma (V600E) and Erdheim–Chester disease. It is classified as L01EC01 (BRAF inhibitors) and was the first-in-class BRAF inhibitor, demonstrating durable responses in melanoma and rare histiocytic disorders. Nevertheless, limitations include resistance, paradoxical MAPK activation, and restricted activity to V600E-mutant cancers.

**Table 4:** Approval status, indications, and clinical considerations for PPP2CA and BRAF inhibitors.

Drug	Approval Status	Approved/ Investigated Indications	Regulatory Classification (ATC)	Clinical Strengths	Limitations
LB-100	Investigational (Phase I/II trials)	Glioblastom, ovarian clear cell, CRC, MDS (trials only)	Antineoplastic agent Enzyme inhibitors	Novel mechanism; broad dependency; potential synergy with chemo & immunotherapy	Systemic toxicity risk (targets common essential gene); not selective; early stage [45]
Dabrafenib	FDA/EMA/MHRA approved (2013)	Melanoma (V600E/K), NSCLC (V600E), thyroid cancer, pediatric glioma, solid tumours	L01EC02—BRAF inhibitors	Precision oncology; highly effective for BRAF-mutant tumours; strong combination data (trametinib)	Resistance via MAPK reactivation; not useful in wild-type BRAF or CRC
Vemuraf- enib	FDA/EMA/MHRA approved (2011–2012)	Melanoma (V600E), Erdheim-Chester disease	L01EC01—BRAF inhibitors	First-in-class BRAF inhibitor; durable responses in melanoma and rare histiocytic disorders	Resistance; paradoxical MAPK activation; only effective in V600E mutation

#### 3.4.2 Identification of Potential Combination with Imatinib

**Figure 9:**
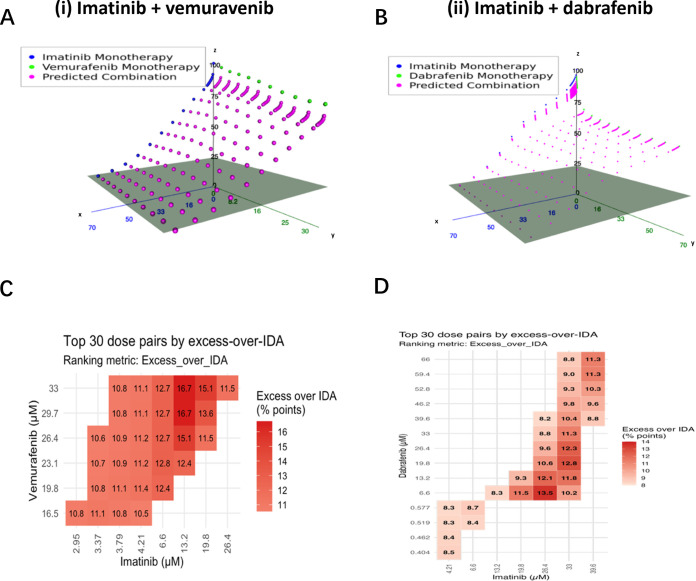
Predicted combination activity of imatinib with vemurafenib and dabrafenib using CTRPv2 monotherapy data. All doses are in μM. (**A,B**): 3-D Independent Drug Action (IDA) surfaces: blue points trace imatinib single-agent viability across doses; green points trace the partner drug (vemurafenib (**A(i)**) or dabrafenib (**B(ii)**). Axes: x = imatinib (μM), y = partner (μM), z = % viability (0–100). Magenta points mark the IDA baseline. For each dose pair, the height of the magenta point on the z axis equals the predicted % viability (the minimum of the two single-agent viabilities). Points positioned lower on the *z*-axis (closer to the grey plane) represent lower predicted viability (greater killing) under IDA. Note: magenta color intensity is fixed and does not encode magnitude. (**C**): Heatmap shows the top 30 dose pairs for imatinib and vemurafenib ranked by excess-over-IDA, where excess-over-IDA = (IDA-predicted viability − observed combination viability); tile numbers are percentage-point gains beyond IDA (higher number/intense red = more benefit). (**D**): Heatmap shows the top 30 dose pairs for imatinib and dabrafenib ranked by excess-over-IDA; tile numbers are percentage-point gains beyond IDA. (see Supplementary Table S5 for full IDACombo results of top 30 dose pairs of imatinib plus dabrafenib).

The potential combination of candidate inhibitors with imatinib was evaluated using the IDACombo platform. For LB-100, no cell line data were available, and therefore the analysis focused on the BRAF inhibitors dabrafenib and vemurafenib, as shown in Fig. 9.

Across 129 haematopoietic/lymphoid cancer cell lines, the combination of imatinib + vemurafenib displayed a broad mid-dose synergy region beyond independent drug action (IDA)(see Supplementary Table S4 for full IDACombo results of top 30 dose pairs). The hotspot centered on imatinib doses of approximately 6.6–13.2 μM combined with vemurafenib at approximately 20–33 μM, yielding an excess-over-IDA of 16–17 percentage points, with many adjacent dose pairs ≥10 pp. At higher imatinib doses, the benefit diminished, consistent with limited additive effect once imatinib alone approached maximal activity. The corresponding IDA 3-D surface revealed a pronounced trough, highlighting a large baseline “gap” that the interaction could exploit, concordant with the heatmap profile.

## 4 Discussion

The analysis of gene differentiation using single-cell transcriptomics provides a valuable approach to investigate gene expression changes across different stages of CML [16]. In this study, the analysis focused on genes within the RAS/RAF signalling pathway, comparing normal donors with CML patients at diagnosis, CML patients before and after TKI treatment, and the persistence of *BCR::ABL1*^*+*^ cells in CML patients following TKI therapy relative to normal donors. Within the predefined set of 48 RAS/RAF pathway–related genes, serine/threonine phosphatase 2A (PPP2CA) was the only gene showing consistent upregulation across all three comparisons. Additionally, BRAF was consistently upregulated in CML patients, suggesting a potential role in *BCR::ABL1*^*+*^ persistence. These results support PPP2CA and BRAF as candidate genes for targeted intervention in patients with persistent CML cells.

PPP2CA encodes the α-isoform and major catalytic subunit of protein phosphatase 2 (PP2A), a serine/threonine phosphatase that regulates processes such as survival, apoptosis, mitosis, DNA repair, and dephosphorylation [50,51]. Interestingly, PPP2CA demonstrates context-dependent functions: at low expression levels, it acts as an essential survival gene, whereas at higher expression levels, it functions as a tumor suppressor [51]. As a core component of PP2A, PPP2CA exerts tumor-suppressive activity through multiple signaling pathways. It functions to inhibit the phosphorylation-driven activation of JAK3 and STAT5 under IL-2 stimulation [51,52]. Similarly, within the RAS/RAF/MEK/ERK pathway, PP2A interacts with ERK2 and MEK1 and indirectly with RAS and RAF, all of which are well-characterised in malignant transformation of susceptible cells. Notably, inhibition of PP2A has been shown to increase hyperactivation of RAS signaling and stabilize c-Myc during the G2 phase, thereby driving mitosis through cyclin E (Cdk1 complexes) [50,53,54]. This mechanism is critical in overcoming treatment resistance. Taken together, these findings align with the hypothesis that PPP2CA is a candidate gene consistently upregulated across cell mutations and expressed higher on the CML cell lines such as KCL22, K562, and KU812, highlighting its role in disease persistence and therapeutic resistance.

PP2A classically acts as a tumour suppressor but it has also been found to be effective at killing leukemic progenitors by promoting SHP-1 tyrosine phosphatase-dependent BCR-ABL1 inactivation/degradation in patient with BC-CML [55,56]. However, emerging evidence from other malignancies-mentioned above, indicates that transient PP2A inhibition can paradoxically enhance therapeutic sensitivity by destabilizing compensatory signaling circuits, particularly when used in combination regimens. This strategy has been reported to sensitize cancer cells to therapeutic stress, enhance apoptosis, and disrupt adaptive survival signalling, particularly when used in combination [45,57]. Accordingly, PPP2CA is described in this study as a conditional therapeutic vulnerability rather than a universal oncogenic driver. Its targeting should be interpreted in a context-specific and combinatorial framework, with careful consideration of dose, timing, and cellular dependency. These findings highlight the complexity of PP2A/PPP2CA biology and underscore the need for further mechanistic validation when exploiting PP2A/PPP2CA modulation as a therapeutic strategy in CML.

LB-100, a first-in-class endothall derivative to reach clinical evaluation, exhibits anticancer activity by selectively inhibiting PP2A/PPP2CA. The compound, formally named *3-(4-methylpiperazine-1-carbonyl)-7-oxabicyclo [2.2.1]heptane-2-carboxylic acid*, shows direct cytotoxicity against tumour cells [45,57]. Evidence from preclinical investigations demonstrated that LB-100 exhibits antitumor activity against nasopharyngeal carcinoma, glioblastoma, breast cancer, sarcoma, hepatocellular carcinoma, pheochromocytoma, pancreatic and ovarian cancer [50]. Importantly, LB-100 has been shown to enhance efficacy without a corresponding increase in toxicity. Further research has indicated that LB-100 functions as a catalytic inhibitor of PP2AC (PPP2CA/PPP2CB), suggesting that its antitumour effects may result from additive suppression of PP2A activity [58]. Currently, the drug is being evaluated in a Phase I clinical trial, with promising initial outcomes [45]. Although LB-100 demonstrates broad activity across diverse cancer models, this study indicate heightened PPP2CA dependency in CML, particularly under TKI treatment pressure. This suggests that LB-100, despite its potential systemic toxicity as an inhibitor of a common essential gene, may hold greater therapeutic value in TKI-treated CML, where its effects could be more selective. While no DepMap data are currently available regarding cell line expression, gene dependency, or drug sensitivity for LB-100, the evidence supports a hypothetical role for this compound in acting synergistically with imatinib to target persistent *BCR::ABL1*^*+*^ cells in CML patients. Nevertheless, further investigation is required to confirm this potential and to elucidate the underlying mechanisms of its activity.

The BRAF gene, located on chromosome 7, encodes an 18-exon cytoplasmic protein that functions as a downstream mediator in the ERK/MAPK signaling cascade, regulating proliferation, differentiation, apoptosis, and cell growth. Alterations in BRAF have been reported in a variety of malignancies, including colorectal cancer, melanoma, brain tumors, papillary thyroid carcinoma, ovarian cancer, and lung cancer [59]. Although BRAF mutations are generally not observed in haematological malignancies, they have been identified and characterised in hairy cell leukemia [59,60]. In study, BRAF was found to be significantly upregulated in patients with CML. Furthermore, on CML-specific cell line, BRAF also demonstrated increased expression, suggesting that BRAF may contribute to disease persistence.

The discovery of oncogenic BRAF mutations has led to the development of small-molecule inhibitors specifically targeting BRAF. Most clinical investigations of BRAF inhibitors have been conducted in metastatic melanoma, where vemurafenib [46] and dabrafenib [61] have demonstrated significant clinical benefit and have subsequently been approved for melanoma treatment. Vemurafenib targets the ATP-binding site of mutant BRAF monomers, thereby suppressing MAPK pathway activation. Early-phase clinical studies demonstrated strong antitumour responses in most melanoma patients, while a Phase III trial reported a 43% overall response rate and significant survival benefit compared with controls. Reported toxicities included arthralgia, fatigue, rash, keratoacanthoma or cutaneous squamous-cell carcinoma, alopecia, nausea, photosensitivity, and diarrhoea [46,59,62]. Dabrafenib, another inhibitor of mutant BRAF, was evaluated in a Phase III randomized clinical trial enrolling patients with advanced or unresectable melanoma. Results showed a clear benefit in progression-free survival, with a median duration of 5.1 months vs. 2.7 months observed in the dacarbazine arm [47]. More than half of the patients developed adverse events of grade 2 or above, predominantly skin-related effects (squamous-cell carcinoma, keratoacanthoma), as well as fever, fatigue, joint pain, and headaches [59].

Evidence from other investigations suggests that low-dose vemurafenib demonstrates strong efficacy in refractory hairy cell leukemia (HCL), with treatment abolishing extracellular signal-regulated kinase (ERK) phosphorylation within hairy cells *in vivo* [63]. In relapsed/refractory HCL, short-term dabrafenib therapy proved both safe and effective, producing objective responses and meaningful clinical benefit across all patients enrolled in a pilot trial, including some previously exposed to vemurafenib. The drug showed anti-leukaemic efficacy comparable to vemurafenib, with the possibility of lower toxicity [49]. The IDAcombo testing of imatinib with vemurafenib or dabrafenib demonstrated a responsive region with notable breadth and peak effect size at specific dose ranges. Given that BRAF inhibitors are optimised for V600E-mutant contexts, whereas CML is typically BRAF wild-type [64], the observed benefit is likely attributable to modulation of the RAF–MEK–ERK signalling cascade or potential network-level off-target effects, which require mechanistic validation. These results indicate the need for further exploration of biological metabolites and associated signalling changes induced by vemurafenib and/or dabrafenib may provide valuable insights into their mechanisms of action and potential therapeutic relevance in CML.

Several limitations should be acknowledged in this study. First, the analyses relied heavily on publicly available datasets (DepMap, DrugBank, PRISM, IDACombo), which, while comprehensive, are limited by variations in experimental platforms, sample representation, and data integration across studies. Second, although PPP2CA and BRAF were systematically evaluated, the functional relevance of their inhibitors remains uncertain in myeloid-derived cell lines, including CML, as none of the candidate drugs (LB-100, dabrafenib, vemurafenib) have yet been directly tested in these contexts. Third, gene dependency scores (Chronos) and expression values were assessed primarily at the transcriptomic and screening level; functional validation in CML models or patient-derived samples was not performed, limiting the translational certainty of the findings. Finally, LB-100 remains in early-phase clinical trials, with incomplete pharmacokinetic, pharmacodynamic, and toxicity profiles, restricting its comparison with clinically established BRAF inhibitors.

## 5 Conclusion

This study highlights PPP2CA as a broadly essential and highly expressed gene in CML-derived myeloid cell lines, with dependency scores indicating stronger essentiality than the median of pan-essential genes. In contrast, BRAF demonstrated moderate and context-specific essentiality, with lower expression and dependency in myeloid models compared with mutation-driven cancers such as melanoma and thyroid carcinoma. The evaluation of inhibitors further underscores these differences: LB-100, a PPP2CA inhibitor currently in early-phase clinical trials, shows broad sensitivity across multiple lineages but remains investigational with limited pharmacological data, while the clinically established BRAF inhibitors dabrafenib and vemurafenib exhibit lineage-restricted activity and well-characterised pharmacokinetic and safety profiles. Collectively, these findings suggest that PPP2CA may represent a novel therapeutic vulnerability in CML, warranting further validation in myeloid-specific preclinical and clinical models, while BRAF inhibitors remain restricted to mutation-enriched malignancies.

## Supplementary Materials





## Data Availability

All data generated or analyzed during this study are included in this published article (and its supplementary information files).

## Supplementary Materials: Supplementary Table S1.

Differentiation result of RNA-seq data of CML patient’s vs. normal donor. **Supplementary Table S2.** Differentiation result of RNA-seq data of CML patient’s after vs. before TKIs treatment. **Supplementary Table S3.** Differentiation result of RNA-seq data of CML patient’s after TKIs treatment vs. normal donor. **Supplementary Table S4**. IDAcombo results: Top 30 dose pairs of imatinib + vemurafenib. **Supplementary Table S5.** IDAcombo results: Top 30 dose pairs of imatinib + dabrafenib. **Supplementary Materials S1**. R-studio script for the differentiation anaylsis. The supplementary material is available online at https://www.techscience.com/doi/10.32604/or.2026.074734/s1.
