# Supplementary material for: Data Mining for Identification of Targets and Repurposed Drugs to Eliminate Persistent Chronic Myeloid Leukaemia Stem Cells: Targeting RAS/RAF Signalling
Source: Oncol Res. 2026 Apr 22;34(5):14. doi: 10.32604/or.2026.074734 (PMC13126564; doi:10.32604/or.2026.074734)
Supplement: Supplementary file 2 [file OncolRes-34-74734-s002.docx]

Supplementary Materials 1. R-studio script for the differentiation anaylisis

1. **Scipt for Deseq2 Patient CP-CML vs Normal**

# ===== 1. Install and Load Required Packages =====

if (!requireNamespace("BiocManager", quietly = TRUE)) install.packages("BiocManager")

if (!require(Matrix)) install.packages("Matrix")

if (!require(data.table)) install.packages("data.table")

if (!require(tidyverse)) install.packages("tidyverse")

# Load libraries

library(Matrix)

library(data.table)

library(tidyverse)

# ===== 2. Set Working Directory =====

# Replace the path below with your own folder where the files are stored

setwd("~/Downloads/Dataset") # <-- change this path as needed

# ===== 3. Read Input Files =====

# Read sparse matrix

counts_sparse <- readMM("E-GEOD-76312.aggregated_filtered_counts.mtx")

# Read gene names (rows)

gene_names <- fread("E-GEOD-76312.aggregated_filtered_counts.mtx_rows", header = FALSE)

# Read sample names (columns)

sample_names <- fread("E-GEOD-76312.aggregated_filtered_counts.mtx_cols", header = FALSE)

# ===== 4. Assign Row and Column Names =====

# Assign gene names as rownames

rownames(counts_sparse) <- gene_names$V1

# Assign sample names as colnames

colnames(counts_sparse) <- sample_names$V1

# ===== 5. Convert to Regular Matrix (Optional, if memory allows) =====

counts_matrix <- as.matrix(counts_sparse)

# ===== 6. Preview the Result =====

# Show first 5 genes and first 5 samples

counts_matrix[1:5, 1:5]

# ===== 7. Save to CSV (Optional) =====

write.csv(as.data.frame(counts_matrix), file = "combined_counts_matrix.csv")

# View entire count matrix in a spreadsheet-like viewer

View(counts_matrix)

print(counts_matrix)

write.csv(as.data.frame(counts_matrix), file = "combined_counts_matrix.csv")

dim(counts_matrix)

# Example output: [1] 18000 12 ← means 18,000 genes and 12 samples

library(Matrix)

library(data.table)

# Read data

counts_sparse <- readMM("E-GEOD-76312.aggregated_filtered_counts.mtx")

gene_names <- fread("E-GEOD-76312.aggregated_filtered_counts.mtx_rows", header = FALSE)

sample_names <- fread("E-GEOD-76312.aggregated_filtered_counts.mtx_cols", header = FALSE)

# Assign row and column names

rownames(counts_sparse) <- gene_names$V1

colnames(counts_sparse) <- sample_names$V1

# Convert and write a subset to Excel (first 100x100)

subset <- as.matrix(counts_sparse[1:100, 1:100])

write.csv(subset, "subset_counts_matrix.csv")

# Load necessary package

library(data.table)

# Read the metadata file (TSV = tab-separated values)

metadata <- fread("ExpDesign-E-GEOD-76312.tsv")

# Check the first few rows

head(metadata)

# Optional: view full metadata in spreadsheet-style tab

View(metadata)

# Subset specific columns with correct column names

metadata_subset <- metadata[, .(Assay, `Sample Characteristic[disease]`)]

# Rename the column

setnames(metadata_subset,

old = "Sample Characteristic[disease]",

new = "Disease")

# Preview the result

head(metadata_subset)

# Remove spaces in names to avoid DESeq warnings

metadata_subset$Disease[metadata_subset$Disease == 'chronic phase chronic myeloid leukemia'] = 'CP-CML'

head(metadata_subset)

# Turn Disease into a factor

metadata_subset$Disease = factor(metadata_subset$Disease, levels=c("normal", "CP-CML"))

metadata_subset$Disease

#Ensure sample names match

rownames(metadata_subset) <- metadata_subset$SampleID # or replace SampleID with the correct column name

counts <- counts_matrix # replace with your object name if different

# ------------------------------------------------------------------

# STEP 2: Prepare DESeq2 dataset

# ------------------------------------------------------------------

if (!requireNamespace("BiocManager", quietly = TRUE))

install.packages("BiocManager")

BiocManager::install("DESeq2")

library(DESeq2)

#check the dimention

dim(counts_matrix) # [genes x samples]

dim(metadata_subset) # should be [samples x variables]

#checking colloum and row name

all(colnames(counts_matrix) %in% rownames(metadata_subset)) # should return TRUE

#Checking what missing

setdiff(colnames(counts_matrix), rownames(metadata_subset))

setdiff(rownames(metadata_subset), colnames(counts_matrix))

#Fixation

colnames(counts_matrix) <- tolower(trimws(colnames(counts_matrix)))

rownames(metadata_subset) <- tolower(trimws(rownames(metadata_subset)))

rownames(metadata_subset) <- metadata_subset$Assay # or whatever your actual ID column is

head(rownames(metadata_subset))

head(colnames(counts_matrix))

all(colnames(counts_matrix) %in% rownames(metadata_subset))

# Run DESeq

# ------------------------------------------------------------------------------

metadata_subset <- as.data.frame(metadata_subset)

rownames(metadata_subset) <- metadata_subset$Assay

metadata_subset <- metadata_subset[colnames(counts_matrix), ]

all(rownames(metadata_subset) == colnames(counts_matrix)) # Should be TRUE

all(rownames(metadata_subset) == colnames(counts_matrix)) # Should be TRUE

metadata_subset <- metadata_subset[colnames(counts_matrix), ]

all(rownames(metadata_subset) == colnames(counts_matrix)) # Should be TRUE now

which(rownames(metadata_subset) != colnames(counts_matrix))[1:10]

rownames(metadata_subset)[1:10]

colnames(counts_matrix)[1:10]

metadata_subset <- as.data.frame(metadata_subset)

# Ensure names are cleaned

metadata_subset$Assay <- tolower(trimws(metadata_subset$Assay))

colnames(counts_matrix) <- tolower(trimws(colnames(counts_matrix)))

# Now set the rownames again

rownames(metadata_subset) <- metadata_subset$Assay

# Then subset and reorder in the correct order

metadata_subset <- metadata_subset[colnames(counts_matrix), ]

# Final check

all(rownames(metadata_subset) == colnames(counts_matrix)) # Should be TRUE

metadata <- fread("ExpDesign-E-GEOD-76312.tsv")

metadata <- as.data.frame(metadata)

metadata$Assay <- tolower(trimws(metadata$Assay)) # Clean sample names

metadata_subset <- metadata[, c("Assay", "Sample Characteristic[disease]")]

colnames(metadata_subset)[2] <- "Disease"

metadata_subset$Disease <- tolower(trimws(metadata_subset$Disease))

metadata_subset$Disease[metadata_subset$Disease == "chronic phase chronic myeloid leukemia"] <- "CP-CML"

metadata_subset$Disease <- factor(metadata_subset$Disease, levels = c("normal", "CP-CML"))

rownames(metadata_subset) <- metadata_subset$Assay

colnames(counts_matrix) <- tolower(trimws(colnames(counts_matrix))) # just in case

metadata_subset <- metadata_subset[colnames(counts_matrix), ] # align order

all(rownames(metadata_subset) == colnames(counts_matrix)) # Should now return TRUE

counts_matrix[1:5, 1:5]

is.integer(counts_matrix)

any(counts_matrix %% 1 != 0) # TRUE means you have non-integers

counts_matrix <- round(counts_matrix)

mode(counts_matrix) <- "integer"

dds <- DESeqDataSetFromMatrix(countData=counts_matrix, colData=metadata_subset, design=~Disease)

# Ignore genes with low counts

dds <- dds[rowSums(counts(dds)) > 10, ]

# Run DESeq

dds <- DESeq(dds)

res <- results(dds, contrast = c("Disease", "CP-CML", "normal"))

summary(res) # Overview

res_ordered <- res[order(res$padj), ]

head(res_ordered) # Top genes

results(dds, contrast=c("disease", "normal", "CP-CML"), alpha=1e-5)

res

system.time({

res <- results(dds, contrast = c("Disease", "CP-CML", "normal"))

})

1. **Scipt for Deseq2 Patient After TKIs vs Normal**

# === 1. Install and Load Required Packages ===

if (!requireNamespace("BiocManager", quietly = TRUE)) install.packages("BiocManager")

BiocManager::install("DESeq2")

install.packages("readr")

install.packages("tidyverse")

install.packages("dplyr")

library(DESeq2)

library(readr)

library(tidyverse)

library(dplyr)

# === 2. Load Full Dataset ===

# Make sure your files are saved in: ~/Downloads/Dataset

setwd("~/Downloads/Dataset")

# Read Excel files from the folder

metadata_full <- readxl::read_excel("Gene Experiment Design.xlsx", sheet = 1)

expression_full <- readxl::read_excel("RAF pathway expression 170825.xlsx", sheet = 1)

# === 3. Preprocess Expression Matrix ===

gene_names <- expression_full$GeneName

counts_matrix <- expression_full %>%

select(-GeneID, -GeneName)

head(counts_matrix)

# Convert to numeric matrix

counts_matrix <- as.data.frame(lapply(counts_matrix, function(x) as.numeric(as.character(x))))

# Add gene names as rownames

rownames(counts_matrix) <- gene_names

# Check structure

str(counts_matrix)

# === 4. Prepare and Filter Metadata ===

metadata_filtered <- metadata_full %>%

select(Assay, `Factor Value[sampling time point]`) %>%

rename(TimePoint = `Factor Value[sampling time point]`) %>%

mutate(TKI_Group = case_when(

TimePoint == "not applicable" ~ "Normal Patient",

TimePoint %in% c("3 month on tyrosine kinase inhibitor treatment",

"6 month on tyrosine kinase inhibitor treatment",

"12 month on tyrosine kinase inhibitor treatment") ~ "After TKIs Treatment",

TRUE ~ NA_character_

)) %>%

filter(!is.na(TKI_Group))

# Convert sample IDs to lowercase for consistency

metadata_filtered$Assay <- tolower(metadata_filtered$Assay)

colnames(counts_matrix) <- tolower(colnames(counts_matrix))

# Subset count matrix to keep only matched samples

counts_matrix <- counts_matrix[, colnames(counts_matrix) %in% metadata_filtered$Assay]

metadata_filtered <- metadata_filtered %>% filter(Assay %in% colnames(counts_matrix))

# Reorder metadata to match count matrix columns

metadata_filtered <- metadata_filtered[match(colnames(counts_matrix), metadata_filtered$Assay), ]

stopifnot(all(metadata_filtered$Assay == colnames(counts_matrix)))

# Set Assay as rownames

rownames(metadata_filtered) <- metadata_filtered$Assay

# Load DESeq2

library(DESeq2)

# Ensure metadata is a data.frame and has rownames

metadata_filtered <- as.data.frame(metadata_filtered)

rownames(metadata_filtered) <- metadata_filtered$Assay

# Check dimensions before running

stopifnot(all(colnames(counts_matrix) == rownames(metadata_filtered)))

# Step 1: Convert all values to numeric (if not already)

counts_matrix[] <- lapply(counts_matrix, function(x) as.numeric(as.character(x)))

# Step 2: Round to nearest integer

counts_matrix <- round(counts_matrix)

# Step 3: Convert to matrix with mode = integer

counts_matrix <- as.matrix(counts_matrix)

mode(counts_matrix) <- "integer"

# Optional: check for decimal leftovers

any(counts_matrix %% 1 != 0) # Should return FALSE

# Create DESeq2 object

dds <- DESeqDataSetFromMatrix(countData = counts_matrix,

colData = metadata_filtered,

design = ~ TKI_Group)

# Filter out low-count genes

dds <- dds[rowSums(counts(dds)) > 10, ]

# Run DESeq

dds <- DESeq(dds)

# === 6. Extract and Save Results ===

res <- results(dds, contrast = c("TKI_Group", "After TKIs Treatment", "Normal Patient"))

# Summary of DESeq2 result

summary(res)

# Add Gene Regulation Column

res_df <- as.data.frame(res)

res_df$Regulation <- ifelse(

res_df$padj < 0.05 & res_df$log2FoldChange > 1, "Upregulated",

ifelse(res_df$padj < 0.05 & res_df$log2FoldChange < -1, "Downregulated", "Not Significant")

)

# Check regulation counts

table(res_df$Regulation)

# Save all significant DEGs (padj < 0.05 & abs(log2FC) > 1)

deg_filtered <- res_df %>% filter(Regulation != "Not Significant")

# Separate upregulated and downregulated

upregulated <- deg_filtered %>% filter(Regulation == "Upregulated")

downregulated <- deg_filtered %>% filter(Regulation == "Downregulated")

# Export results

write.csv(res_df[order(res_df$padj), ], "DEGs_After_TKIs_vs_Normal_patient.csv")

write.csv(upregulated, "Upregulated_genes.csv", row.names = TRUE)

write.csv(downregulated, "Downregulated_genes.csv", row.names = TRUE) # === 1. Install and Load Required Packages ===

1. **Script for DESeq2: Before vs After Treatment**

# === 1. Install and Load Required Packages ===

if (!requireNamespace("BiocManager", quietly = TRUE)) install.packages("BiocManager")

BiocManager::install("DESeq2")

install.packages("readr")

install.packages("tidyverse")

install.packages("dplyr")

library(DESeq2)

library(readr)

library(tidyverse)

library(dplyr)

# === 2. Load Full Dataset ===

# Make sure your files are saved in: ~/Downloads/Dataset

setwd("~/Downloads/Dataset")

# Read Excel files from the folder

metadata_full <- readxl::read_excel("Gene Experiment Design.xlsx", sheet = 1)

expression_full <- readxl::read_excel("RAF pathway expression 170825.xlsx", sheet = 1)

# === 3. Preprocess Expression Matrix ===

gene_names <- expression_full$GeneName

counts_matrix <- expression_full %>%

select(-GeneID, -GeneName)

head(counts_matrix)

# Convert to numeric matrix

counts_matrix <- as.data.frame(lapply(counts_matrix, function(x) as.numeric(as.character(x))))

# Add gene names as rownames

rownames(counts_matrix) <- gene_names

# Check structure

str(counts_matrix)

# === 4. Prepare and Filter Metadata ===

metadata_filtered <- metadata_full %>%

select(Assay, `Factor Value[sampling time point]`) %>%

rename(TimePoint = `Factor Value[sampling time point]`) %>%

mutate(TKI_Group = case_when(

TimePoint == "at diagnosis" ~ "Before TKIs Treatment",

TimePoint %in% c("3 month on tyrosine kinase inhibitor treatment",

"6 month on tyrosine kinase inhibitor treatment",

"12 month on tyrosine kinase inhibitor treatment") ~ "After TKIs Treatment",

TRUE ~ NA_character_

)) %>%

filter(!is.na(TKI_Group))

# Convert sample IDs to lowercase for consistency

metadata_filtered$Assay <- tolower(metadata_filtered$Assay)

colnames(counts_matrix) <- tolower(colnames(counts_matrix))

# Subset count matrix to keep only matched samples

counts_matrix <- counts_matrix[, colnames(counts_matrix) %in% metadata_filtered$Assay]

metadata_filtered <- metadata_filtered %>% filter(Assay %in% colnames(counts_matrix))

# Reorder metadata to match count matrix columns

metadata_filtered <- metadata_filtered[match(colnames(counts_matrix), metadata_filtered$Assay), ]

stopifnot(all(metadata_filtered$Assay == colnames(counts_matrix)))

# Set Assay as rownames

rownames(metadata_filtered) <- metadata_filtered$Assay

# Load DESeq2

library(DESeq2)

# Ensure metadata is a data.frame and has rownames

metadata_filtered <- as.data.frame(metadata_filtered)

rownames(metadata_filtered) <- metadata_filtered$Assay

# Check dimensions before running

stopifnot(all(colnames(counts_matrix) == rownames(metadata_filtered)))

# Step 1: Convert all values to numeric (if not already)

counts_matrix[] <- lapply(counts_matrix, function(x) as.numeric(as.character(x)))

# Step 2: Round to nearest integer

counts_matrix <- round(counts_matrix)

# Step 3: Convert to matrix with mode = integer

counts_matrix <- as.matrix(counts_matrix)

mode(counts_matrix) <- "integer"

# Optional: check for decimal leftovers

any(counts_matrix %% 1 != 0) # Should return FALSE

# Create DESeq2 object

dds <- DESeqDataSetFromMatrix(countData = counts_matrix,

colData = metadata_filtered,

design = ~ TKI_Group)

# Filter out low-count genes

dds <- dds[rowSums(counts(dds)) > 10, ]

# Run DESeq

dds <- DESeq(dds)

# === 6. Extract and Save Results ===

res <- results(dds, contrast = c("TKI_Group", "After TKIs Treatment", "Before TKIs Treatment"))

# Summary of DESeq2 result

summary(res)

# Add Gene Regulation Column

res_df <- as.data.frame(res)

res_df$Regulation <- ifelse(

res_df$padj < 0.05 & res_df$log2FoldChange > 1, "Upregulated",

ifelse(res_df$padj < 0.05 & res_df$log2FoldChange < -1, "Downregulated", "Not Significant")

)

# Check regulation counts

table(res_df$Regulation)

# Save all significant DEGs (padj < 0.05 & abs(log2FC) > 1)

deg_filtered <- res_df %>% filter(Regulation != "Not Significant")

# Separate upregulated and downregulated

upregulated <- deg_filtered %>% filter(Regulation == "Upregulated")

downregulated <- deg_filtered %>% filter(Regulation == "Downregulated")

# Export results

write.csv(res_df[order(res_df$padj), ], "DEGs_After_vs_Before_TKIs_treatment.csv")

write.csv(upregulated, "Upregulated_genes.csv", row.names = TRUE)

write.csv(downregulated, "Downregulated_genes.csv", row.names = TRUE)
